# A Two-Level Cache for Distributed Information Retrieval in Search Engines

**DOI:** 10.1155/2013/596724

**Published:** 2013-11-28

**Authors:** Weizhe Zhang, Hui He, Jianwei Ye

**Affiliations:** School of Computer Science and Technology, Harbin Institute of Technology, P.O. Box 320, Harbin 150001, China

## Abstract

To improve the performance of distributed information retrieval in search engines, we propose a two-level cache structure based on the queries of the users' logs. We extract the highest rank queries of users from the static cache, in which the queries are the most popular. We adopt the dynamic cache as an auxiliary to optimize the distribution of the cache data. We propose a distribution strategy of the cache data. The experiments prove that the hit rate, the efficiency, and the time consumption of the two-level cache have advantages compared with other structures of cache.

## 1. Introduction

With the rapid growth of the Internet users and the increasing query requests, the centralized queries for the same search engine will undermine searching abilities. In addition, it will lead to the increase of the response time and overloading. Caches in the distributed full-text searching have high theory and application value to solve these problems [1]. This is a key technology to improve response time, processing efficiency, and system performance. Caches play an important role in the process of distributed text retrieval.

Melnik et al. [2] design a pipeline to create inverted indexes; it improves the efficiency of generating the indexes. However, such measures just optimize dictionaries, indexes, and other related factors. It has little improvement of the architecture of distributed search engines. It cannot fundamentally solve the problems. However, caches can effectively tackle these problems.

Caches are widely used in various fields of computers and; they can effectively remove the system bottlenecks and enhance the processing ability. Currently, caches have been widely used to improve the performance of search engines. They are a key to enhance the processing ability and shorten the response time for search engines. In recent years, through statistical analysis of user behaviors in search engines, it is found that user queries follow a high degree of repeatability and locality [3]. Lots of Internet users propose a large number of repetitive queries, which are often concentrated in certain local contents. These hot contents can maintain a large number during a certain period. Thus, caches can be used to ease the pressure of search engines, and it is also one of the best ways to improve retrieval performance [4].

This paper proposes a two-level cache structure based on query analysis of user logs, so that search engines can improve the performance of distributed full-text retrieval.  Section 2 describes the related works.  Section 3 proposes a two-level cache structure and explains the design concept, the concrete realization, and their characteristics.  Section 4 shows a distribution strategy of cache data; the last section introduces the theory analysis and experimental verification for the two-level cache structure.

## 2. Related Works

The key techniques of distributed caches include cache topology, data distribution, data synchronization, cache replacement algorithms, cache structures, and stored contents [5].

Among them, the cache structure and stored contents are extremely important in a distributed cache system. Their performance is directly related to the performance of the whole cache system [6]. In a distributed system, the cache replacement algorithms [7] are used when records are written to caches but their storage is full. Then, some data need to be removed from cache storage based on access frequencies, the time intervals, and the last access time. Common cache replacement algorithms include least recently used algorithm (LRU), the minimum frequency of visits algorithm (least frequently used), and recently used algorithm (most recently used). In response to these limitations of the traditional cache replacement algorithms, there are a lot of improved ones. These replacement algorithms are based on the LFU and LRU algorithms. They add some other criteria such as time intervals and access frequencies. They are the deformation of the traditional replacement algorithms. In addition, the document size is introduced to distinguish the characteristics of the replacement algorithms, such as the SIZE algorithm.

Load balancing strategies of distributed caching system are mainly used to solve some cache problems. For example, unbalanced tasks and network congestion will decrease the process ability. Cache systems are mainly used to dynamically balance the tasks in the different servers, so as to improve system performance and speed, and to provide better access quality. Now there are polling, minimum connection number, and process ability balance strategies [8].

## 3. Two-Level Cache Architecture

According to the analysis of hot words and repeatability of queries, it has been found that previous hot spots will still be repeated and maintained hot in current user queries. Based on this phenomenon, if the hot words are initialized in the caches, the entire cache hit rate can be improved. This paper designs a two-level cache based on user query logs and integrates it into a distributed caching system.

### 3.1. Cache Structure

The structure of the cache design is as follows. In each cluster, each cache server adopts a two-level cache structure. The caches consisted of a static cache and a dynamic cache. The static and dynamic caches work together to cache data and enhance the processing speed. In entire cache structure, static cache will work at first and then dynamic cache does. When each query arrives, our system will look it up in the static cache. If it hits, the cached data is returned; otherwise, it accesses dynamic cache to see if it hits. According to different situation, cache system uses different processes. The static cache stores the highest frequency queries and retrieves the results. By analyzing the query logs in the cluster, the hottest queries are extracted, and the corresponding search results are in pairs stored in the static cache. Data in static cache are relatively fixed. The contents will change only when the static cache needs to rebuild and replace some contents in the cache. The dynamic cache changes according to user queries; it stores high frequency user queries and retrieval results. The store is dynamic. After a period of time, with the help of replacement algorithm, the contents in dynamic storage are also relatively hot.

The two-level cache structure mainly consists of the following four parts.

*(a) Creating a Static Cache and Dynamic Cache with Memory Buffer.* To provide the high speed of the cache system, all cache servers are using the memory as a buffer. Cache operating module in each server will create a static cache and a dynamic cache. The sizes of the static cache and dynamic cache are based on the actual requirement; this work will test with different cache capacity and give performances evaluation. Specifically, operation of creating different caches is completed by an open source software called Ehcache. 

*(b) Initializing Static and Dynamic Caches with the Data Distribution Strategy.* By analyzing the previous day query log of the cluster, we can calculate the number of different query requests, the last query time, query time interval, and the survival of the query. According to the formula for calculating the hot values, the system will count the hot values of each query. Sorted by the hot values, the first few queries and their corresponding results will be stored in the static cache. The details will be introduced in the next section. The dynamical cache is empty at first. There is no data in it. In the whole cache system, each server not only will store the native hot queries and corresponding results but also will store the data from other servers in the same cluster. So the cache system will communicate with cache systems in other servers.

**Figure 1:**
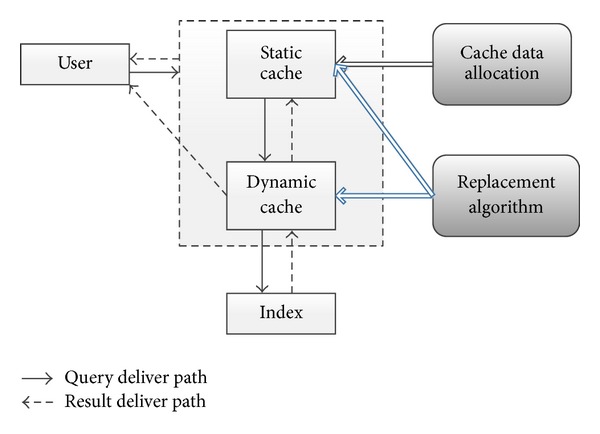
Collaborative mechanism of static and dynamic caches.

*(c) Designing Coordination Mechanism between Static and Dynamic Caches.* Static and dynamic caches constitute a cache structure. The two kinds of caches work together to enhance search engines performance. The collaborative mechanism of the static and dynamic caches is shown in Figure 1.

After the creation of static cache and dynamic cache, the cache data allocation module will initialize the value in data in the cache. When the initialization is completed, the cache system can start to work to process user queries. With the arrival of each query, our system will look it up in the static cache. If the query is hit in the cached data, this data is returned; otherwise, it accesses dynamic cache to see if the query is hit. If the query is missing in both static and dynamic caches, the query will be processed in the cluster. When returning the results to the user, the static and dynamic caches will exchange some records with the replacement algorithm.

*(d) Updating the Static and Dynamic Caches. *The system uses synchronous buffer initialization strategy to update the indexes. The system updates its indexes every 24 hours. Before the update, the indexes in the cluster will not change. When the index is updated, cache system will destroy the static cache and dynamic cache. Then, it will initialize the data of static cache and dynamic cache based on the allocation strategy to ensure consistency of cache and index data.

### 3.2. Characteristics of Two-Level Cache

Two-level cache has static and dynamic caches, which have the following characteristics.

- The contents stored in the static cache are relatively fixed, in addition to part of the replacement of cache replacement algorithm; the majority of its content does not change within 24 hours. The static cache is updated daily and the update is based on the query log generated every day in this cluster. After the index updates, by analyzing the query log, we can extract the hot query to allocate the data of the static cache.
- The data in static cache are from analyzing the query logs, which provide the hot queries and corresponding results for the static cache. By the query repeatability analysis, the popular content stored in the static cache will still be popular in the next 24 hours. Thus, the static cache hit rate should be high; it enhances the whole cache hit rate.
- For static cache, the content is nearly consistent in a day, and it uses the synchronous buffer initialization strategy with the update of index. It avoids the data flow caused by keeping the data synchronization in all the cache servers.
- The dynamic cache acts as an adjunct to static cache, whose data change dynamically. We will record the dynamic cache, check if the buffer is full, and write caches. Otherwise, you will replace the adjustment in accordance with the replacement strategy and static cache and dynamic cache records.

## 4. Cache Data Distribution Strategy

The cache data distribution strategy refers to how the queries and its corresponding results will be stored in the two-level cache structure cache. The entire cache data distribution strategy includes the following content: how to calculate the hot value of every query from the query logs, during the initialization of static cache and dynamic cache, how to decide which queries and the corresponding results to be stored in the static cache, and how to replace the data in static cache and dynamic cache.

The idea of the strategy is very simple. By analyzing the cache queries log, the cache system will calculate the hot value of every query. Then, the cache system will choose the top *N* queries sorted by their hot values. Finally, the system will put these queries and their corresponding results in the static cache. At the same time, in order to achieve further optimization expansion, the cache will also store the hot queries and their corresponding results in other clusters. This needs communication among the cache servers. During the work of the caching system, the data in the static cache and dynamic cache are updated with the cache replacement algorithm.

**Figure 2:**
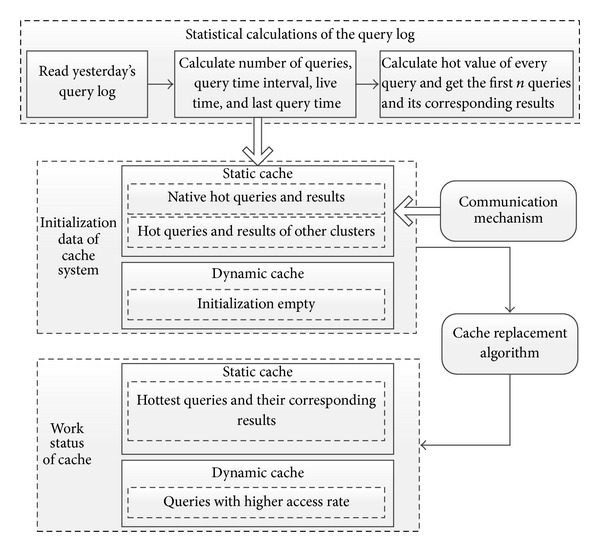
Distribution strategy of static cache and dynamic cache data.

Cache data allocation strategies can be divided into the following parts: calculating of the query log, cache data initialization, the communication between different cache servers about hot queries, and data exchange between static and dynamic caches. The cache data distribution strategy is shown in Figure 2.

Implementation of cache data distribution strategy is as follows.

*(a) Analyzing the Query Log and Calculating the Hot Value of Every Query.* Analyzing the query log is the first step. Then, we calculate the hot value of every query. The next step is to open the query log file, read the query log contents, and extract every query item. We calculate the total times of queries, the first time of query, and the last time of query. We know that the query with high frequency has greater hot value. However, getting hot spot just based on query times and query frequency may get a past hot spot; the users are less likely to query the requests. Thus, two characteristics are introduced into the paper; they are *Query Life-Cycle* and *Query Inactive Time*. *Query Inactive Time* is the current system time minus the last query appearing in the query log.

First, we calculate the query frequency; the query with higher frequency is more likely to be hot content. Putting these queries in the cache will increase the hit rate. Because the cache structure based on log is built on web collection system, the log will be generated every day. When calculating the query frequency, the system will run 24 hours; therefore, the query frequency is calculated as follows:

(1) $$\begin{array}{c} \text{Freg}=\frac{\text{QueryNum}}{24*3600}. \end{array}$$

In the formula, QueryNum is the number of queries times.

The interval time is calculated as follows:

(2) $$\begin{array}{c} \text{IntervalTime}=\frac{1}{\text{Freq}}. \end{array}$$

*Query Life-Cycle* is the time between the first occurrence of a query and the last occurrence, which is calculated as follows:

(3) $$\begin{array}{c} \text{LiveTime}=\text{LastTime}-\text{FirstTime}. \end{array}$$

In the formula, LastTime is the time of its last occurrence and FirstTime is the time of its first occurrence.

To ensure the accuracy of hot content after statistical calculating, the system introduces a characteristic called NotActiveTime. NotActiveTime is the current system time minus the last query in the query log. NotActiveTime is calculated as follows:

(4) $$\begin{array}{c} \text{NotActiveTime}=\text{CurrentTime}-\text{LastTime}. \end{array}$$

In the formula, CurrentTime is current time and LastTime is the last time of the query.

After statistical calculating, for each query, we can get its query frequency *Freg*, its live time LiveTime, and its not active time NotActiveTime. The hot value of a query is proportional to Freg and LiveTime, and it is inversely proportional to NotActiveTime. The system calculates the hot value based on these three variables. And HotValue is calculated as follows:

(5) $$\begin{array}{c} \text{HotValue}=\text{Freq}*\text{LiveTime}*\frac{1}{\text{NotActiveTime}}. \end{array}$$

With the above formula of HotValue, the system will calculate the hot value of every query and then sort the queries by HotValue in descending order.

*(b) Initialization of Static Cache and Dynamic Cache Ache.* Data initialization can be divided into two parts: one is static cache data initialization and the other is dynamic cache data initialization. Dynamic cache data initialization is very simple. The system just needs dynamic cache area. It has no data in the dynamic cache, and then its data keep changing with the user query behavior. Static cache initialization is relatively harder, which includes the following two parts.

1. Put the native hot queries and their corresponding results into the cache.
2. Put the hot queries and their results from other clusters into the cache.

In the native cluster, after the statistical calculating, the cache system will get hot value of each query and then sort these queries by hot value in descending order. Then, choose the first *N* queries and search their corresponding results from the system. Then, put these queries and the results into the static cache. After that, the initialization of native static cache is finished.

After native initialization, the system is going to initialize data from other clusters; the system will collect hot queries and their corresponding results from off-site clusters and then put them in its static cache. It is mainly completed with communication and read-write mechanism between clusters. Off-site clusters get their hot queries with the same method mentioned above, and then each off-site cluster will share its first *M* queries with highest hot value and their corresponding results with other clusters. Finally, the system will get first *M* queries from all the other clusters by communication. Assume that there are *X* clusters in the cache system in total. Then finally there are *N* + *M*∗(*X* − 1) records in static cache of a cluster.

*(c) Communication between Static Cache and Dynamic Cache.* After the initialization of static cache, one cluster has *N* + *M*∗(*X* − 1) records in its static communication. This initialization data is based on the query log, by statistical analysis and HotValue calculating. We finally get the hot queries and their corresponding results. According to the consistency of hot content, most of the content will maintain a good access rate today. However, hot content of yesterday may plummet today, and these contents may have low query rate. In view of this situation, the system introduces a mechanism about how to dynamically adjust the data of static cache and dynamic cache. And it is realized with cache replacement algorithm.

## 5. Experiments

The cache structure has superior performance and higher hit rate; there are the two following reasons.

- Using static cache, after initialization, hot queries and their corresponding results are stored in static cache. When the user queries come, most of them will match the data in cache. Compared with just using dynamic cache, it needs a period of time to make the hit rate from low to high. Therefore, using two-level cache structure will have a higher hit rate.
- The cache is made of memory, and thus it is fast. However, as the cache size increases, although the hit rate increases, the processing speed of finding a record will be slower. For a cache server, it is not large cache capacity which makes better performance. The cache structure of the system on the whole can be divided into two parts. Static cache initialized with hot queries based on query log and its corresponding results. With the help of cache replacement algorithm, the content in static is the hottest queries. For each query, the system first looks it up in static cache.

In this paper, experimental data based on query log are used to compare the cache server with two-level cache structure.

Hardware environment is as follows: desktop with 4 cores, AMD Athlon (tm) II CPU 2.6 G, 250 G hard disk and 2 GRAM. The number of query requests is, respectively, as follows: 1000 2000 5000 10000 20000 30000 50000 80000. The experiment is using two different cache structures, so there are two test cases: Case 1 is using the two-level cache structure and Case 2 just uses dynamic cache.

In the experiments, the cache in two-level cache structure is divided into two halves: 500 records in static cache and 500 records in dynamic cache. But cache with just dynamic cache holds all of the 1000 records. Static cache in two-level structure will not change and the dynamic cache changes with LRU algorithm. The dynamic cache structure also changes with LRU algorithm.

*Test 1.* Test two-level cache structure and pure dynamic cache 2 cases. For different total queries, the result of single query processing time is shown in Table 1. 

*Test 2.* Test the hit rates of 2 cases; the results are shown in Table 2.

From the test results in Tables 1 and 2, some conclusion can be made. Two-level cache structure based on query log puts the hottest queries and their corresponding results into the static cache. After the initialization, 500 records in static cache may have a high access frequency. Compared with using only the dynamic cache, the performance of two-level cache structure is much higher than only using dynamic cache at start stage. When the total queries are 1000, 2000, 5000, and 10000, the processing speed is better than only using dynamic cache. But with the increase of queries number, the advantage of the two-level cache structure is no longer obvious. When the total queries are 50000, 80000, or more, its performance is nearly the same with structure of the dynamic cache caching system. After analyzing cache records, the records in static cache are fixed in a period of time, but this data may not be hot today. And it is contrary to our design ideas. After analyzing the hit rate of static cache and dynamic cache, it was found that the hot content based on the query log can only represent popular queries in the past, access frequency of some of the queries may decrease, and, what is worse, the access frequency of few queries may be low, affecting the performance of the two-level cache structure. However, this is not saying that the two-level cache structure design is not scientific, and designing a reasonable cache replacement algorithm can solve this problem. In order to maintain the heat and freshness of a static cache, the design is not only to achieve cache replacement but also to achieve replacement between static cache and dynamic cache.

## 6. Conclusions

The paper introduces a two-level cache structure based on query log; the cache is divided into two parts, static cache and dynamic cache. The system initializes its data after analyzing the query log, with the help of replacement algorithm. The static cache stores the hottest queries and their corresponding results. Compared with just using dynamic cache, the two-level cache structure initializes its data by query logs. Thus, at the beginning of the system, the hit rate can be maintained in a high level. The whole cache is composed of static cache and dynamic cache. Cache matching (look up query in cache) can be fast. The experimental results show that two-level cache structure is 28% faster than dynamic cache.

## Figures and Tables

**Table 1:** The processing time of single query.

Total queries (times)	Processing time of single query by using two-level cache structure (ms)	Processing time single query by just using dynamic cache (ms)
1000	32.3	59.6
2000	31.6	51.2
5000	39.5	54.5
10000	46.3	48.6
20000	53.1	50.3
30000	55.2	52.6
50000	50.8	50.4
80000	49.1	48.7

**Table 2:** The number of hits while dealing with different total queries.

Total queries (times)	Hit counts of two-level cache structure (times)	Hit counts of just using dynamic cache (times)
1000	354	208
2000	694	451
5000	1332	1025
10000	2135	2093
20000	5214	5526
30000	7198	7663
50000	11210	12329
80000	19522	20861
